# Development and Validation of a Scale for Measuring Leadership and Managerial Competencies of Middle Managers in Health Care and Medical Education in the Gulf Region: Cross-Sectional Study

**DOI:** 10.2196/77476

**Published:** 2026-06-04

**Authors:** Ahmed Mohamed Al Ansari, Elizabeth L Holloway, Reginald Paul Sequeira

**Affiliations:** 1 National Health Regulatory Authority, 1011, Manama, 11464, Bahrain, 973 36788880; 2 Royal College of Surgeons in Ireland - Bahrain, Muharraq, Bahrain; 3 Graduate School of Leadership & Change, Antioch University, Yellow Springs, Ohio, United States; 4 Department of Pharmacology & Therapeutics, College of Medicine and Health Sciences, Arabian Gulf University, Manama, Bahrain

**Keywords:** leadership, managerial competency, medical education, health care, management, middle management, lower management, scale development, middle managers

## Abstract

**Background:**

The Kingdom of Saudi Arabia and the Kingdom of Bahrain are transforming their health care systems toward more self-sustained, autonomous systems. Effective leadership at all management levels, particularly middle management, is critical for operational success.

**Objective:**

The study aimed to develop a feasible, reliable, and valid scale for measuring leadership and managerial competencies of middle managers in health care and medical education in the Gulf region.

**Methods:**

A mixed methods approach was adopted. Semistructured interviews were conducted with top management, middle management, and lower management staff (N=27). Reflexivity of coders and thematic saturation were considered to ensure the reliability and transparency of qualitative findings. Thematic analysis informed scale item creation. A total of 202 participants from medical education and health care sectors completed the scale. Cronbach α, exploratory factor analysis (principal axis factoring), and confirmatory factor analysis validated internal consistency and factor structure. Responses marked as “6=unable to assess” were treated as missing data during quantitative analysis.

**Results:**

Seven themes emerged: personality, agility, attitude, managerial skills, work ethics, mental ability, and interaction. Three constructs were identified: professionalism and problem-solving, team management and adaptation, and time management and expertise. A validated scale with 17 competency measure points and 16 characteristic items was finalized. Only competency measure points underwent exploratory factor analysis and confirmatory factor analysis, while characteristic items were retained for descriptive evaluation.

**Conclusions:**

The Leadership and Managerial Competency Scale for Middle Managers in the Gulf Region reflects perceived competencies valued at all management levels. The scale measures perceived importance, not actual performance; the conclusions align with the validated scope.

## Introduction

Globally, the health care sector is experiencing significant transformation as more effective and efficient models of care delivery are developed in response to pressing challenges. Health care systems in the Gulf region face the challenge of delivering effective responses to issues such as communicable diseases, rapid population growth, a rising older population, socioeconomic reforms, liberal urbanization, and changes in lifestyle, which reflect on the health determinants of the population [1-3]. The Kingdom of Saudi Arabia and the Kingdom of Bahrain are currently transforming their health care systems from a government system to a more self-sustained autonomous system [1,2]. This goal requires effective leadership at all management levels, especially at the middle management level, which is vital for effective planning and operational implementation [4,5].

Leadership has been discussed from diverse perspectives such as philosophical, managerial, and psychological [3,6,7]. Key themes identified across leadership definitions include the idea that leaders work toward attaining a shared objective [8,9], leaders unify and influence rather than dictate, and the results of leadership constitute an improved current state [10,11]. Leaders are involved in the selection, equipping, training, and influencing of followers, who themselves possess a collection of diverse skills, talents, and abilities [12-15]. Managerial perspectives discuss leadership roles in critical organizational functions such as strategy execution and resource use [15-17]. In the health care sector, efficient leadership is central to improvements as organizations seek to improve quality and efficiency in health care [18,19], improve the management of human resources, achieve turnover and cost reductions, as well as fulfill specific strategic priorities [13,20-22]. Leadership in health care is also tasked with functions such as addressing the disempowerment of marginalized groups, improving accountability, fostering transparency and cross-communication, encouraging innovations, and expanding awareness of pertinent clinical and administrative issues [13,23,24]. Preparation for health care leadership is currently embedded in the training of physicians [21,25,26]. The leadership components prepare doctors to handle complex leadership and management scenarios, the evolving needs of stakeholders in the health care sector, and various external factors that they might encounter in the workplace and throughout their careers [9,14,20,27-30].

Mid-level management is in the middle of hierarchical managerial systems, situated between top-level managers and managers of the lowest (first) level. Mid-level managers are responsible for the implementation of top management decisions; they interpret the strategy set by top-level management and oversee its implementation [3,31,32]. They are authorized to manage the organizational units and are responsible for the results of these units. The role of the middle manager includes directing personnel, administering resources, operations control, and solving complex issues within organizations [4,33-35]. Effective middle management is critical for the success of the organizations, as the agency is responsible for translating strategies into successful operations and achieving high-performance cultures [5,24,36,37]. Middle managers play a central role in implementing health care innovation, particularly by addressing the informational gaps between senior management and frontline clinicians, mediating between strategy and day-to-day activities, and building momentum for innovation implementation [33,36,38]. Successful leaders and middle managers in the health care sector must therefore possess a complex set of skills across several areas, including clinical, leadership, managerial, social, and personal aspects [8,26,39-41].

Several studies emphasize the connection between transformational leadership and managerial behavior, in general, and middle management, in particular [42]. Such leaders communicate a well-articulated vision, create a sense of belonging, encourage employees to adjust positively to changes, and provide coaching to subordinates [23,42-45]. Other intersecting competencies identified for middle managers in the literature include cultural competency, the ability to effectively manage a workforce comprised of individuals from multiple cultural and ethnic groups and adapt to ways of doing business in new cultures and environments [40,46], learning-oriented leadership and at-work learning [41], and high emotional intelligence [47].

Arab leadership forms the underlying framework for health care leadership in the Middle East. One of the main differences between Arab leadership and other kinds of leadership (Western leadership) is that the Arab leadership is based on the Quran and accepted Hadiths [48,49]. In the Quran, God provides people with principles, tools, and skills that are required in leading their lives, relating with others, and in realizing their fullest potential. Arab leaders are not free to act as they choose or must submit to the wishes of others but must only act to implement the laws of Allah on earth [48,50]. Thus, the motivational agencies in this style of leadership derive from spiritual sources. Arab leadership is characterized by spiritual and religious behavior and is oriented toward people rather than products [1,2,48,50]. Arab leadership (Islamic leadership) is transformational, as leaders actively work to facilitate and enable transformation in their followers in tandem with Islamic values [49]. As in other cultural contexts, in Arab contexts, middle managers often oversee the implementation of quality improvement and innovative initiatives within their facilities, including innovations in health care [32,36,39], all while working within the tenets of Islamic values. Developments, such as increasing complexities in health financing, new institutional models, changes in regulation, insurance models, challenges in assuring patient safety and high-quality care, as well as challenges in meeting strategic priorities, have now made it urgent to consider the key competencies that characterize successful second-line leaders in the health care sector [39,51-53]. This study evaluated the competencies required for effective middle management in health care institutions and medical educational institutions in the kingdoms of Saudi Arabia and Bahrain. The leadership and managerial competencies identified in the study were then applied to construct a scale for required competencies for middle managers in health care and medical education in the Gulf region.

## Methods

### Study Design

The study was implemented using a mixed methods approach involving both a qualitative and a quantitative phase. The qualitative phase involved 3 distinct steps: the collection of primary data using semistructured interviews; the analysis of the data using emergent thematic analysis; and the conversion of the outputs from data analysis into scale items. Scale development involved developing a reliable and validated measure of the construct such that an attribute of interest was measured using an appropriate numerical dimension [54]. This study followed the 5-step mixed method proposed by Zhou [55] as the conceptual framework for scale development.

### Step 1: Qualitative Exploration

Semistructured interviews, using a validated interview guide, were conducted to develop items for the leadership and managerial competencies scale. The interview guide was developed by the research team and is provided as Multimedia Appendix 1. Participants were selected using a simple, modified random sampling technique. The inclusion criteria were that participants must be 30 years of age or older, work in an educational or health care organization with distinct management roles, and work in an educational or health care organization in the Kingdom of Bahrain or in the Kingdom of Saudi Arabia.

The sample (N=27) comprised 7 top leaders (CEO, dean, or vice dean), 10 middle managers (heads of the clinical departments in a hospital setting and heads of departments in an academic setting), and 10 employees (working under middle managers). In the study, 37% (n=10) of the participants were in the 31 to 40 years age group, 51% (n=14) were in the 41 to 50 years age group, 7% (n=2) were in the 51 to 60 years age group, and 3% (n=1) were in the 61 to 70 years age group. Therefore, the data obtained largely reflect the perceptions of managers who are between 41 and 50 years of age. The majority of middle managers were within the 35 to 50 years age group (n=4, 15%). A total of 62% (n=17) of the sample were male participants, and 37% (n=10) were female participants. The mean years of experience in the present position of the sample related to top leadership, middle, and lower management in health care was 6 (SD 4.2) years.

### Step 2: Thematic Analysis and Converting Qualitative Data to Scale Items

The data from step 1 were analyzed using an emergent thematic analysis approach to identify competency and characteristic scale items. The competency scale items were referred to as competency measure points (CMPs). A team of researchers, each with expertise in qualitative research and leadership competencies, independently coded the interview transcripts to identify initial themes and subthemes. Each researcher was assigned a distinct portion of the data to code, ensuring various perspectives and reducing the risk of bias in the analysis. The coding process followed a structured procedure, with each researcher highlighting the key excerpts from the interviews that represented relevant themes or subthemes.

After initial coding, the researchers met to compare and discuss their findings. A series of team discussions was held to review the codes and the themes that emerged from them. Any discrepancies or disagreements between the coders were resolved through team discussions, in which the team aimed to reach a consensus on the themes and subthemes. This process was critical to ensure the reliability and consistency of the themes identified. Thematic saturation was monitored throughout the qualitative analysis. Saturation was considered to be reached when no new themes or meaningful variations in existing themes emerged from subsequent interviews. During coding, the research team engaged in regular discussions to reflect on how their professional backgrounds in health care leadership and medical education might influence the interpretation of the data, ensuring themes remained grounded in participant perspectives.

The final themes were refined through iterative discussions and re-examination of the data until all researchers agreed on the final list of themes. These final refined themes and subthemes were then converted into scale items. The scale items were framed as clear, concise questions that reflected the competencies identified in the thematic analysis.

The scale items were developed using a 5-point Likert scale to measure the level of importance (1=not at all important, 2=less important, 3=neutral, 4=important, and 5=very important) [7]. To ensure validity, the scale items were reviewed for clarity and relevance, and any ambiguous items were modified.

### Step 3: Content Validation

The face validity and content validity of the scale items were assessed by sending the newly developed scale to 6 experts for an independent review. These experts were senior professionals in health care management and leadership, with extensive experience in research and scale development. Their expertise included organizational leadership, hospital administration, medical education, and human resource management. The items were presented as a binomial scale with 2 options to denote whether the corresponding item could be included in the scale or not: (1) “Yes” or “No” and (2) “Agree” or “Disagree.” Any disagreements among the experts were resolved through a follow-up discussion, during which the team of researchers reviewed the feedback provided and addressed any concerns raised by the experts. A consensus approach was used to determine which scale items to retain; items were selected for inclusion in the scale if 4 experts out of 5 (80%) or 5 experts out of 6 (>83%) agreed on their inclusion [9]. In cases where further clarification was needed, additional rounds of discussion were held to ensure the items’ appropriateness. The newly developed and validated scale is presented in MultimediaAppendices 2  3, respectively. A total of 19 CMPs and 16 characteristic scale items were developed.

### Step 4: New Scale Administration

In the Kingdom of Bahrain, 2 medical universities and 3 main public hospitals were approached by the principal investigator through email and telephonic conversations. Similarly, 10 of the largest universities and hospitals were approached through email and telephonic conversations in the Kingdom of Saudi Arabia. After obtaining consent from the universities and hospitals, the developed scale items (MultimediaAppendices 2  3, respectively) were administered to willing participants. To ensure that the scale could capture diverse perspectives, demographic variables such as age, gender, nationality, and origin were included in the survey. These variables were collected to explore potential variations in responses based on different participant characteristics. Specifically, we analyzed whether there were any significant differences in the important ratings of leadership and managerial competencies across various demographic groups. The survey was sent to 500 individuals, with 202 responding to the survey. This response rate was adequate as the target number of respondents was 150. Responses marked as “6=unable to assess” were treated as missing values and excluded from statistical analyses. The data from the survey were analyzed using SPSS version 19 [11].

### Step 5: Quantitative Validation (Reliability and Construct Validity)

Exploratory factor analysis (EFA) and confirmatory factor analysis (CFA) were conducted only on the CMPs, which represent core competency constructs. Characteristic items, which reflect personal and interpersonal qualities, were retained for descriptive evaluation but were not included in the factor analysis. Reliability is a measure of the internal consistency of the measuring instrument [4]. Cronbach α was used to check the internal consistency of the instrument by calculating the α coefficient [10]. Construct validity was confirmed using EFA and CFA, ensuring that the identified CMPs accurately reflected distinct competency dimensions. EFA using principal components extraction with varimax rotation was conducted to identify the underlying competency dimensions represented in the scale.

### Ethical Considerations

The research protocol, including both qualitative and quantitative data collection, was approved by the review boards of the participating hospitals and medical educational settings in the Kingdoms of Saudi Arabia (REC-HSD-B-1‐2020) and Bahrain (E025-PI-12/20). All ethical principles required for studies involving human participants were upheld. All participants signed informed consent before participating in the study. All procedures followed were in accordance with the ethical standards of the responsible committees on human experimentation (institutional and national) and with the Declaration of Helsinki of the World Medical Association.

Informed consent was obtained from all individual participants involved in the study. Participants were fully informed of the nature and possible consequences of the research before providing their consent.

To protect participant privacy and confidentiality, all data were anonymized and deidentified prior to analysis. No identifying information (eg, names, hospital numbers, or direct identifiers) was collected or reported. Access to raw data was restricted to the research team and stored on secure servers in compliance with local data protection regulations. No compensation was provided to participants for taking part in this study.

## Results

### Participant Demographics

A total of 27 leaders and managers were interviewed in the step 1 survey, and 202 participants were involved in the step 4 survey. Most of the qualitative data obtained were from middle and lower management staff, reflecting their key role in the research focus.

### Qualitative Findings: Key Themes and Subthemes

Seven key themes representing competencies and skills that are critical for middle managers emerged from the thematic analysis (step 2) of the qualitative data: personality, agility, attitude, managerial skills, work ethics, mental ability, and interaction. Each of these key themes was further refined into subthemes, providing a deeper understanding of the competencies required.

- Personality: self-confidence, emotional stability, and adaptability
- Agility: decision-making under pressure, responsiveness to change, and strategic flexibility
- Attitude: proactiveness, resilience, and willingness to learn
- Managerial skills: delegation, leadership style, and communication effectiveness
- Work ethics: accountability, integrity, and commitment
- Mental ability: critical thinking, problem-solving, and analytical reasoning
- Interaction: team collaboration, negotiation skills, and conflict resolution

Through EFA (step 5), 3 interrelated constructs were identified that encapsulate the core competencies required for middle management in the Gulf region:

- Team management and adaptation
- Professionalism and problem-solving
- Time management and expertise

### Quantitative Findings

Based on the findings from the qualitative analysis and subsequent review by the expert panel, a set of survey items was developed for measuring leadership and managerial competencies of middle managers in the Gulf region (Kingdom of Bahrain and Kingdom of Saudi Arabia), as shown in MultimediaAppendices 2  3. The survey was distributed to 500 individuals, and 202 responses were received, resulting in a 40.4% response rate, which exceeded the target of 150 respondents. Descriptive statistics, including the percentage of responses, mean and SD, and measures of skewness and kurtosis, were computed for each item in the scale.

### Exploratory Factor Analysis

A principal component analysis with varimax rotation for the CMPs was carried out using the original 19 items in Multimedia Appendix 2. The determination of the number of factors to be extracted depends on how strongly and cleanly the variables load on the factors. The variable will load strongly on a particular factor if the loading is greater than 0.40, and it is considered clean if the absolute difference between the loadings is greater than 0.20. Factor analysis results for the competency scale demonstrated that CMP11 (“Should be an effective communicator”) and CMP13 (“Should be organized in thoughts, words, and deeds”) were removed due to low factor loadings (<0.40) and poor alignment with other items, resulting in a more robust and interpretable factor structure. Therefore, CMP11 and CMP13 were deleted from the model (Table 1). Varimax rotation was applied to achieve a clearer factor structure by maximizing the variance of factor loadings. Although moderate correlations between factors were observed, the orthogonal rotation facilitated the identification of distinct competency domains during the exploratory stage of analysis.

**Table 1.** Exploratory factor analysis of competency scale item^a^.

Serial number	Scale	Item	Factor 1	Factor 2	Factor 3	Commonalities
1	Should be able to work in a team	CMP1^b^	—^c^	0.776	—	0.64
2	Should be able to work with multiple teams	CMP10	0.569	—	—	0.64
3	Should be an effective listener	CMP12	0.618	—	—	0.69
4	Should demonstrate integrity	CMP14	0.841	—	—	0.75
5	Should display effective organizational skills	CMP15	0.761	—	—	0.71
6	Should follow democratic ways	CMP16	0.545	—	—	0.57
7	Should have long-term vision for the organization	CMP17	0.754	—	—	0.59
8	Should possess leadership skills	CMP18	0.788	—	—	0.64
9	Should possess problem-solving skills	CMP19	0.681	—	—	0.72
10	Able to adapt to changes	CMP2	—	0.694	—	0.71
11	Should be able to achieve tasks as per timeline	CMP3	—	—	0.770	0.63
12	Possess up-to-date knowledge in the respective field	CMP4	—	—	0.590	0.63
13	Should address any conflict of interest effectively	CMP5	—	—	0.573	0.61
14	Should be able to balance various roles (multitasking)	CMP6	—	0.550	—	0.69
15	Should be able to encourage teamwork	CMP7	—	0.773	—	0.68
16	Should be able to manage resources (money or manpower)	CMP8	—	0.728	—	0.72
17	Should be able to take appropriate decisions	CMP9	0.662	—	—	0.72

a Kaiser-Meyer-Olkin measure and Bartlett’s test of sphericity. Kaiser-Meyer-Olkin measure of sampling adequacy is 0.96 in this study. Approximate *𝛘*2_136_=2905.11 and significance at 0.

b CMP: competency measure point.

c Not applicable.

The Bartlett test of sphericity in EFA evaluated all the factors together and each factor separately against a hypothesis stating that there are no factors. The Bartlett test of sphericity in this study was significant (*P*<.001), indicating that enough shared variance is present. The Kaiser-Meyer-Olkin (KMO) is a measure that provides an approach to comparing the zero-order correlations with the partial correlations between pairs of variables. The KMO in the study model was 0.96. Kaiser (1974) stated that if the KMO is greater than 0.50, it is acceptable. The closer the KMO value is to 1, the better the correlations between the pairs of variables that can be explained by the other variables.

Analysis of moment structure (AMOS) implements the general approach to data analysis known as structural equation modeling. The multivariate normality of the data was examined by conducting normality checks using the AMOS (version 24.0) software. The distribution of the 17 items in this study is acceptable because none of them deviates from normality (Table 1). Cronbach α coefficient for the internal consistency of the total sample was 0.93. The Cronbach α value was found to be 0.94 for factor 1, 0.93 for factor 2, and 0.86 for factor 3.

A structural model was identified for the scale based on 3 interrelated constructs—professionalism and problem-solving, team management and adaptation, and time management and expertise. As stated previously, these 3 factors emerged from the EFA analysis. The level of correlation between the variables was determined, and then the statistical model fit was evaluated for alignment with the actual dataset using a number of “goodness-of-fit” statistics, as presented in Table 2.

**Table 2.** Correlation matrix.

Factor	Professionalism and problem-solving	Team management and adaptation	Time management and expertise
Professionalism and problem-solving	1.000	0.791	0.759
Team management and adaptation	0.791	1.000	0.773
Time management and expertise	0.759	0.773	1.000

### Confirmatory Factor Analysis

The CFA was carried out on the variance-covariance matrix for the 3-factor model through the AMOS (version 24.0) statistical package. The estimation method used was maximum likelihood. To achieve model identification, the regression coefficients of the error terms over the endogenous variables were fixed to 1. The CFA was carried out to determine whether the hypothesized statistical model fit the actual dataset, and a number of “goodness-of-fit” statistics were used on the 3-factor models derived by means of EFA. The model fits the data adequately, with a goodness of fit index (0.90), comparative fit index (0.90), Tucker-Lewis index (0.96), and root mean square of error approximation (0.06). The raw *χ*^2^ is 116, and the χ^2^/*df* is 1.76, with a *P* value less than .001.

The final validated scale developed through these processes is termed the Leadership and Managerial Competency Scale for Middle Managers in the Gulf Region (LMCS-MM Gulf Region; Multimedia Appendix 4) and reflects what people in the 3 levels of management (lower, middle, and top) value.

## Discussion

### Main Findings

The objective of this study was to develop a valid scale for measuring the competencies for successful middle managers in health care and medical education in the Gulf region, particularly in the Kingdom of Saudi Arabia and the Kingdom of Bahrain. Despite the recognized importance of middle management in health care, there has been a notable gap in the availability of validated instruments specifically tailored to the cultural and operational contexts of the Gulf region. This study aimed to fill that void.

Scholars proffer that interviews can facilitate the collection of key information regarding a variable of interest, and that the findings from the analysis of such data in turn inform the survey design and final scale development [52,56]. This study followed the above principle and adopted the 5-step mixed method by Zhou [55] as the conceptual framework for scale development. The framework involves qualitative exploration, scale item identification, content validation, scale data collection, scale administration, and scale validation [55].

In step 1, qualitative interviews examined the scale construct of interest, which is the competency required for successful middle management. Data were collected based on the personal and social contextual and developmental characteristics that are relevant for successful middle managers, providing the foundation for scale item identification and the evidence to support the content validity of the new scale. The competencies explored in the interviews were extracted from the empirical literature and were consistent with the previous frameworks, such as Goleman emotional intelligence model and Bass transformational leadership theory, indicating theoretical convergence. A number of studies identified transformational leadership attributes such as a well-articulated vision, the ability to create a sense of belonging, providing coaching to subordinates, and inspiring or motivating behavioral change in followers as being essential for success as a middle manager [23,42-45]. Competencies such as emotional intelligence, cultural competency, promoting learning cultures, and high emotional intelligence [24] were also identified in the literature and align with the findings from the qualitative interviews conducted in this study. Numerous authors have observed that one of the ways through which strategic priorities and improvements in operational results can be obtained in the health care sector is through leveraging leadership development [6,13,38,57]. Skills and qualities such as leadership, empathy, effective decision-making, communication, collaboration, and community leadership are emphasized in medical education with a view to instilling these values into future medical professionals [27,53,56,58]. These findings reinforce the construct validity of the scale and reflect cross-contextual alignment with widely accepted competency frameworks.

In step 2, themes were converted to codes and then to scales, transforming the qualitative data into measurable items on ordinal scales that would allow the use of CFA for data analysis. In step 3, a review of the scale items for content validity was done using debriefing and panel review. The expert panel selected 19 items that should be included in the survey. In step 4, the new instrument was administered to a large representative sample as a quantitative survey.

In step 5, evidence of construct-based validity of the scale items was established through a series of statistical tests measuring scale validity and reliability. Scale validity involves testing the new scale to ascertain that it indeed measures the construct that it is designed to measure (valid scale), and scale reliability means that the scale measures the construct in a precise and consistent manner [54,59]. A scale can be valid but not reliable, and vice versa. Therefore, both measures of quality are important aspects in the scale development process to ensure the accuracy and adequacy of the scale [60,61].

Reliable scales yield the same results every time the underlying condition is the same. Thus, reliability indicates that the scale is consistent [55,62]. Internal consistency reliability was measured for the new scale and focuses on the consistency between different items. It indicates the extent to which participants in the study rated the multiple-item construct measure administered to them in a similar manner [51,60]. For this scale, reliability was estimated in terms of average item-to-total correlation, and Cronbach α was calculated. Cronbach *α* is a measure of reliability that measures the strength of consistency between different items. The resulting reliability coefficient α has a value ranging from 0 to 1. If scale items are not related or correlated, then *α*=0.

The factors that emerged from the EFA using principal axis factoring were professionalism and problem-solving, team management and adaptation, and time management and expertise. These resulting constructs from the EFA were validated using CFA. The internal consistency of the factors was measured using composite reliability. Good reliability was obtained as the coefficient values were greater than 0.70.

Assessment of validity using empirical methods yields a type of validity known as criterion-related validity [58,63]. Thus, criterion-related validity was measured for the new scale. In this study, the convergent and discriminant validity of the new scale were also measured. Convergent validity indicates how closely a measure converges or relates to the construct being measured, while discriminant validity shows the opposite effect or discrimination.

Bivariate correlations were computed for this 3-item measure of competencies for the successful middle manager—professionalism and problem-solving, team management and adaptation, and time management and expertise. Adequate discriminant validity was obtained for the scale on the competencies of middle managers, as the average variance extracted was greater than the maximum shared squared variance. For convergent validity, the average variance extracted was 0.50 or higher and lower than the composite reliability. In other words, the variance explained by the construct was greater than the measurement error and greater than cross-loadings. Thus, a validated scale for measuring the competencies required for successful middle managers in the Gulf region emerged from EFA and CFA, with statements related to the characteristics and competencies required for middle managers in the Gulf region.

It is important to highlight that the scale developed under this study (LMCS-MM Gulf Region scale) did not measure the skills and attributes of potential candidates for middle management positions. Rather, the measures reflect what people in the 3 levels of management (lower, middle, and top) value in a (hypothetical) middle manager. Many of the items found in the scale, such as emotional intelligence, transformational leadership (intellectual stimulation and individualized consideration), high-level cognitive skills, and a constellation of other skills (decision-making, communication, problem-solving), already have existing, reliable measurement tools, further strengthening both the reliability and the use of the scale for measuring leadership and managerial competencies. The competencies identified should also be interpreted within the cultural and organizational context of the Gulf region. Middle managers frequently serve as key intermediaries by translating strategic decisions into operational practice within the relatively hierarchical institutional structures. Workplace cultures in the region often emphasize collaboration, interpersonal respect, and relationship-based leadership. These contextual characteristics may have contributed to the prominence of competencies such as teamwork, integrity, adaptability, and effective interpersonal interaction observed in the present scale. The identified competency domains also align with the global health care leadership frameworks, such as the NHS Healthcare Leadership Model, which emphasizes teamwork, professional credibility, ethical leadership, and effective decision-making. This alignment suggests that while leadership competencies may be influenced by local cultural context, many core competencies remain consistent across international health care systems.

Nevertheless, the study has limitations. The sample was restricted to the 2 Gulf countries, which may limit generalizability across the entire Middle East and North Africa region. Additionally, while expert validation was conducted, end-user feedback from actual middle managers in practice was not captured in the final validation stage. These factors may implicate how the scale proves to be universally applicable or context-sensitive. Future research should focus on applying this scale across different health care systems and cultural settings in the wider Arab world to evaluate its external validity. Longitudinal studies could also examine how scores on the scale predict actual performance outcomes or leadership development trajectories in middle managers.

From a practical perspective, organizations can use the LMCS-MM Gulf Region scale for structured recruitment, leadership development, and performance evaluation. Embedding this scale into managerial training programs could help in aligning individual growth with institutional leadership expectations and foster sustainable health care leadership pipelines. As the data were collected using a single self-report survey instrument, the possibility of common method variance cannot be entirely excluded. Future studies may employ multisource assessments or longitudinal designs to reduce this potential bias and further validate the scale.

### Conclusion

This study developed and validated the LMCS-MM Gulf Region scale, offering a robust tool to assess essential competencies in health care and medical education. The scale demonstrates strong psychometric properties and addresses a critical gap in leadership assessment within the Gulf context. The scale measures the perceived importance of leadership and managerial competencies rather than directly assessing managerial performance. As such, it provides a structured framework that may support leadership development, training, and competency-based management initiatives within health care and medical education institutions. Its application can enhance recruitment, training, and performance evaluation of middle managers, thereby supporting the ongoing health system transformations and leadership development initiatives in the region.

## Supplementary material

10.2196/77476 Multimedia Appendix 1 The use of semistructured interviews.

10.2196/77476 Multimedia Appendix 2 Competency scale items developed after content validation.

10.2196/77476 Multimedia Appendix 3 Characteristic scale items developed after content validation.

10.2196/77476 Multimedia Appendix 4 Final scale developed: Leadership and Managerial Competency Scale for Middle Managers in Gulf Region (LMCS-MM Gulf Region).

## Abbreviations

AMOS: analysis of moment structure
CFA: confirmatory factor analysis
CMP: competency measure point
EFA: exploratory factor analysis
KMO: Kaiser-Meyer-Olkin
LMCS-MM Gulf Region: Leadership and Managerial Competency Scale for Middle Managers in Gulf Region
