# Supplementary material for: Development and Validation of a Scale for Measuring Leadership and Managerial Competencies of Middle Managers in Health Care and Medical Education in the Gulf Region: Cross-Sectional Study
Source: JMIR Med Educ. 2026 Jun 4;12:e77476. doi: 10.2196/77476 (PMC13235977; doi:10.2196/77476)
Supplement: Multimedia Appendix 1 [file mededu-v12-e77476-s001.docx]

**Supplemental file 1:** The Use of Semi-Structured Interviews*.*

To gain participants’ thoughts on middle manager skills and characteristics, the following questions were used in the interview:

1. Tell me about your personal and professional journey. How long have you worked as a middle manager and what have been your experiences?
2. Can you describe an individual whom you thought was an excellent middle manager? (with special focus on personal traits)
3. Can you tell me the social qualities this person possessed to become a successful middle manager?
4. In your opinion, what do you think are the competencies of this successful middle manager that might have played a greater role in overall growth of a University / Health care organization in the Gulf region (Kingdom of Bahrain and Kingdom of Saudi Arabia)?
5. In your opinion, what do you think are the competencies possessed by this successful middle manager, for effective leadership in a University / Health care organization in the Gulf region (Kingdom of Bahrain and Kingdom of Saudi Arabia)?
6. In your opinion, what do you think are the competencies of this successful middle manager, which would have contributed to his / her development in a University / Health care organization in the Gulf region (Kingdom of Bahrain and Kingdom of Saudi Arabia)?

Each interview lasted for about 15 to 20 minutes. All interviews were held virtually through the ZOOM platform and scheduled based on mutually agreed time slots. All interviews were recorded after getting informed consent from the participants.
