# Supplementary material for: Development and Validation of a Scale for Measuring Leadership and Managerial Competencies of Middle Managers in Health Care and Medical Education in the Gulf Region: Cross-Sectional Study
Source: JMIR Med Educ. 2026 Jun 4;12:e77476. doi: 10.2196/77476 (PMC13235977; doi:10.2196/77476)
Supplement: Multimedia Appendix 2 [file mededu-v12-e77476-s002.docx]

*Table S1- Competency scale items developed after content validation:*

In your opinion, “What are the characteristics that are critical or most significant for a successful Middle Manager?” (Chairman / Heads of the Departments / Heads of the Clinical Services) should possess?" Please select the appropriate option on a scale of 1 to 5 (*(1 = Not at all important, 2 = less important, 3 = Neutral, 4 = important, 5 = very important)* If you are not sure of the item, please select “6” (unable to assess) for that item.

| **S.NO** | **Competency Scale items** | **1** | **2** | **3** | **4** | **5** | **6** |
| --- | --- | --- | --- | --- | --- | --- | --- |
| 1 | Be able to work in a team |  |  |  |  |  |  |
| 2 | Able to adapt to changes |  |  |  |  |  |  |
| 3 | Be able to achieve tasks as per timeline |  |  |  |  |  |  |
| 4 | Possess up- to-date knowledge in the respective field |  |  |  |  |  |  |
| 5 | Should address any conflict of interest effectively |  |  |  |  |  |  |
| 6 | Should be able to balance various roles (multitasking) |  |  |  |  |  |  |
| 7 | Should be able to encourage teamwork |  |  |  |  |  |  |
| 8 | Should be able to manage resources (money /manpower) |  |  |  |  |  |  |
| 9 | Should be able to take appropriate decisions |  |  |  |  |  |  |
| 10 | Should be able to work with multiple teams |  |  |  |  |  |  |
| 11 | Should be an effective communicator |  |  |  |  |  |  |
| 12 | Should be an effective Listener |  |  |  |  |  |  |
| 13 | Should be organized in his/her thoughts, words and deeds |  |  |  |  |  |  |
| 14 | Should demonstrate integrity |  |  |  |  |  |  |
| 15 | Should display effective organizational skills |  |  |  |  |  |  |
| 16 | Should follow democratic ways |  |  |  |  |  |  |
| 17 | Should have long term vision for the organization |  |  |  |  |  |  |
| 18 | Should possess leadership skills |  |  |  |  |  |  |
| 19 | Should possess problem-solving skills |  |  |  |  |  |  |
