# Supplementary material for: Development and Validation of a Scale for Measuring Leadership and Managerial Competencies of Middle Managers in Health Care and Medical Education in the Gulf Region: Cross-Sectional Study
Source: JMIR Med Educ. 2026 Jun 4;12:e77476. doi: 10.2196/77476 (PMC13235977; doi:10.2196/77476)
Supplement: Multimedia Appendix 3 [file mededu-v12-e77476-s003.docx]

*Table S2- Characteristic scale items developed after content validation:*

In your opinion, “What are the competencies that are critical or most significant for a successful Middle Manager (Chairman / Heads of the Departments / Heads of the Clinical Services)? Please select the appropriate option on a scale of 1 to 5 (*(1 = Not at all important, 2 = less important, 3 = Neutral, 4 = important, 5 = very important)* If you are not sure of the item, please select “6” (unable to assess) for that item

| **S.NO** | **Characteristics scale items** | **1** | **2** | **3** | **4** | **5** | **6** |
| --- | --- | --- | --- | --- | --- | --- | --- |
| 1 | Be a motivator |  |  |  |  |  |  |
| 2 | Be accessible to all stakeholders |  |  |  |  |  |  |
| 3 | Be approachable all the time |  |  |  |  |  |  |
| 4 | Be productive |  |  |  |  |  |  |
| 5 | Be supportive to all stakeholders |  |  |  |  |  |  |
| 6 | Have desire to grow |  |  |  |  |  |  |
| 7 | Have more self-confidence |  |  |  |  |  |  |
| 8 | Should manage time efficiently |  |  |  |  |  |  |
| 9 | Should be active always |  |  |  |  |  |  |
| 10 | Should be available for guidance |  |  |  |  |  |  |
| 11 | Should be corruption free |  |  |  |  |  |  |
| 12 | Should be humble |  |  |  |  |  |  |
| 13 | Should display patience towards subordinates and superiors |  |  |  |  |  |  |
| 14 | Treat everyone with respect |  |  |  |  |  |  |
| 15 | Willing to learn from others |  |  |  |  |  |  |
| 16 | Willing to share ideas with others |  |  |  |  |  |  |
