# Supplementary material for: Development and Validation of a Scale for Measuring Leadership and Managerial Competencies of Middle Managers in Health Care and Medical Education in the Gulf Region: Cross-Sectional Study
Source: JMIR Med Educ. 2026 Jun 4;12:e77476. doi: 10.2196/77476 (PMC13235977; doi:10.2196/77476)
Supplement: Multimedia Appendix 4 [file mededu-v12-e77476-s004.docx]

*Table S3- Final scale developed: “Leadership and Managerial Competency Scale for Middle Managers in Gulf Region (LMCS-MM Gulf Region)”*

| S.NO | Item Description | 1 | 2 | 3 | 4 | 5 | 6 |  |
| --- | --- | --- | --- | --- | --- | --- | --- | --- |
| Competency measure points (CMPs) | | | | | | | | |
| 1 | Be able to work in a team |  |  |  |  |  |  |  |
| 2 | Able to adapt to changes |  |  |  |  |  |  |  |
| 3 | Be able to achieve tasks as per timeline |  |  |  |  |  |  |  |
| 4 | Possess up-to-date knowledge in the respective field |  |  |  |  |  |  |  |
| 5 | Should address any conflict of interest effectively |  |  |  |  |  |  |  |
| 6 | Should be able to balance various roles (multitasking) |  |  |  |  |  |  |  |
| 7 | Should be able to encourage teamwork |  |  |  |  |  |  |  |
| 8 | Should be able to manage resources (money / manpower) |  |  |  |  |  |  |  |
| 9 | Should be able to take appropriate decisions |  |  |  |  |  |  |  |
| 10 | Should be able to work with multiple teams |  |  |  |  |  |  |  |
| 11 | Should be an effective Listener |  |  |  |  |  |  |  |
| 12 | Should demonstrate integrity |  |  |  |  |  |  |  |
| 13 | Should display effective organizational skills |  |  |  |  |  |  |  |
| 14 | Should follow democratic ways |  |  |  |  |  |  |  |
| 15 | Should have long term vision for the organization |  |  |  |  |  |  |  |
| 16 | Should possess leadership skills |  |  |  |  |  |  |  |
| 17 | Should possess problem-solving skills |  |  |  |  |  |  |  |
| Characteristic scale items | | | | | | | | |
| 18 | Be a motivator |  |  |  |  |  |  |  |
| 19 | Be accessible to all stakeholders |  |  |  |  |  |  |  |
| 20 | Be approachable all the time |  |  |  |  |  |  |  |
| 21 | Be productive |  |  |  |  |  |  |  |
| 22 | Be supportive to all stakeholders |  |  |  |  |  |  |  |
| 23 | Have desire to grow |  |  |  |  |  |  |  |
| 24 | Have more self-confidence |  |  |  |  |  |  |  |
| 25 | Should manage time efficiently |  |  |  |  |  |  |  |
| 26 | Should be active always |  |  |  |  |  |  |  |
| 27 | Should be available for guidance |  |  |  |  |  |  |  |
| 28 | Should be corruption free |  |  |  |  |  |  |  |
| 29 | Should be humble |  |  |  |  |  |  |  |
| 30 | Should display patience towards subordinates and superiors |  |  |  |  |  |  |  |
| 31 | Treat everyone with respect |  |  |  |  |  |  |  |
| 32 | Willing to learn from others |  |  |  |  |  |  |  |
| 33 | Willing to share ideas with others |  |  |  |  |  |  |  |
